# Evaluation of Reminder App for Optimization of Bladder Filling Status During Hypo-Fractionated Irradiation for Prostate Cancer: Protocol of REFILL-PAC-HYPO Trial

**DOI:** 10.3390/clinpract15030040

**Published:** 2025-02-20

**Authors:** Dirk Rades, Jan-Dirk Küter, Michael von Staden, Ahmed Al-Salool, Christian Ziemann, Stefan Janssen, Julia Koeck, Justus Domschikowski, Charlotte Kristiansen, Christine Vestergård Madsen, Marciana N. Duma, Tobias Bartscht, Jon Cacicedo, Florian Cremers

**Affiliations:** 1 Department of Radiation Oncology, University Medical Center Schleswig-Holstein, Lübeck Campus, 23538 Lübeck, Germanyflorian.cremers@uksh.de (F.C.); 2 Radiation Oncology Department, University of the Basque Country, 48903 Barakaldo, Vizcaya (Basque Country), Spain; 3 Medical Practice for Radiotherapy and Radiation Oncology, 30161 Hannover, Germany; 4 Department of Radiotherapy, Malteser Hospital St. Franziskus, 24939 Flensburg, Germany; 5 Department of Oncology, Vejle Hospital, University Hospital of Southern Denmark, 7100 Vejle, Denmark; 6 Department of Radiotherapy, Helios Hospital Schwerin, 19055 Schwerin, Germany; 7 Department of Hematology, Oncology and Stem Cell Transplantation, Helios Hospital Schwerin, 19055 Schwerin, Germany; 8 Radiation Oncology Department, Hospital Universitario Cruces, 48903 Barakaldo, Vizcaya (Basque Country), Spain; 9 Radiation Oncology Department, Biobizkaia Health Research Institute, 48903 Barakaldo, Vizcaya (Basque Country), Spain

**Keywords:** prostate cancer, hypo-fractionated radiation therapy, bladder filling status, radiation cystitis, reminder app

## Abstract

**Background/Objectives:** During radiotherapy for prostate cancer, the risk of radiation cystitis is increased if the volume of the bladder is small. According to previous studies, it is important that bladder volumes are ≥200 mL. Drinking protocols may be helpful in this context. Adherence to such protocols can be challenging, and may be improved by an app reminding patients before each session of radiotherapy to drink a certain amount of water. Our prospective phase 2 trial (REFILL-PAC-HYPO, NCT06784115) evaluates the impact of a reminder app on bladder filling in prostate cancer patients treated with hypo-fractionated radiotherapy. **Methods:** Twenty-seven patients need to be recruited for the REFILL-PAC-HYPO trial. Radiotherapy, preferably with volumetric-modulated arc therapy, uses hypo-fractionation with 20 × 3.0 Gy over four weeks. An app reminds patients to drink water (300 mL) 45 min before each session of hypo-fractionated irradiation. On the last day of their treatment, patients are asked about their satisfaction with the app. In the case of a dissatisfaction rate of >20%, the app requires modifications. If this rate is >40%, the app is considered not useful. Additionally, patients are asked about the impact of their participation in the trial and using the app on their general attitude towards health technology. Furthermore, the phase 2 cohort is compared to a historical control group treated with hypo-fractionated radiotherapy during recent years but not supported by an app. The REFILL-PAC-HYPO trial will contribute to identifying the potential value of a reminder app for bladder filling during hypo-fractionated radiotherapy for prostate cancer.

## 1. Introduction

Many patients with prostate cancer, which is a very common type of cancer, are treated with radiotherapy, either as external beam radiotherapy (EBRT) alone, brachytherapy alone, or a combination of EBRT and brachytherapy [1,2]. Since three randomized trials have demonstrated the non-inferiority of moderately hypo-fractionated EBRT (dose per fraction from >2.0 to 3.4 Gy and overall treatment time from 4 to 5.5 weeks) when compared to normo-fractionated radiotherapy (dose per fraction of 2.0 Gy and overall treatment time from 7.5 to 8 weeks), moderately hypo-fractionated radiotherapy has become more popular, particularly for patients with low or intermediate prostate cancer [3,4,5,6,7]. Two of the three randomized trials used 20 × 3.0 Gy for HF-RT [4,5]. Radiotherapy for prostate cancer, irrespective of the type of fractionation, may lead to significant acute toxicity, including radiation cystitis. It was previously reported that a smaller bladder volume of <200 mL (or <180 mL) led to a higher rate of urinary toxicity [8,9,10]. Furthermore, a planned bladder volume of >200 mL resulted in decreased motion of the prostate during the corresponding radiation sessions [11]. In another study, the recommended dose constraints for the bladder were achieved less frequently if its volume was <200 mL [12]. Thus, it is important that bladder volumes are ≥200 mL during most or even all radiation sessions.

In a recent study, we investigated the bladder volumes in 76 patients receiving moderately hypo-fractionated EBRT alone with 20 × 3.0 Gy, at each of the 20 fractions [13]. The mean and median numbers of radiation fractions with bladder volumes of <200 mL were significantly (*p* < 0.001) higher in the subgroup of patients with bladder volumes of <200 mL prior to the course of moderately hypo-fractionated EBRT. These results indicate a medical need for an improvement in bladder filling status during moderately hypo-fractionated EBRT, especially in patients with bladder volumes of <200 mL prior to the start of the radiation course [13].

Other groups have evaluated the value of drinking protocols where patients were requested to drink water before computed tomography (CT) simulation and each treatment session [10,11,12,14,15,16,17,18,19,20,21,22,23,24]. The amount of water was between 200 and 600 mL and the interval between water intake and CT simulation or daily radiation was between 30 and 60 min [10,11,12,14,15,16,17,18,19,20,21,22,23,24]. Following a drinking protocol may require substantial discipline from patients. Therefore, we decided to create a mobile application (app) that reminds patients before a session of radiotherapy to drink water. The REFILL-PAC-HYPO trial investigates a number of radiation sessions with bladder volumes of <200 mL in a prospective series of patients treated with moderately hypo-fractionated EBRT alone for localized prostate cancer using such a reminder app. In addition, the REFILL-PAC-HYPO trial evaluates whether the use of the reminder app leads to a significant reduction in the proportion of radiotherapy sessions with bladder volumes of <200 mL when compared to a historical control group not supported by a reminder app.

## 2. Experimental Design and Materials

### 2.1. Objectives and Endpoints

The main objective of the REFILL-PAC-HYPO trial is the evaluation of the effect of an app reminding patients receiving moderately hypo-fractionated radiotherapy for localized prostate cancer to drink water before each session of radiotherapy on the number of sessions during which the bladder volume was <200 mL. The app reminds patients to drink 300 mL of water 45 min before each radiotherapy session. The selection of these parameters was based on clinical experience and previous studies [10,11,12,14,15,16,17,18,19,20,21,22,23,24]. When considering these studies, the median time interval between water intake and radiotherapy was 45 min, and, therefore, this was selected for the REFILL-PAC-HYPO trial. Since the median amount of water was 400 mL, this amount was initially chosen for the trial. However, the centers participating noted that a considerable number of patients irradiated for prostate cancer experienced difficulties in retaining urine until the completion of their radiation session if they drank more than 300 mL before the session. Therefore, an amendment of the protocol using 300 mL instead of 400 mL was submitted to and approved by the Ethics Committee. The primary endpoint of the REFILL-PAC-HYPO trial is the number of radiotherapy sessions with a bladder volume of <200 mL at the end of treatment (20 fractions). The secondary endpoints include satisfaction with the app and the impact of participation in the trial and using the app on general attitudes towards health technology. The patients’ adherence to the app is assessed. The patients have to confirm their intake of water each day and receive a puzzle piece as a reward. Moreover, the patients are interviewed by staff members during their daily visits to the radiotherapy department regarding their time and amount of water intake.

### 2.2. General Trial Design and Duration

The REFILL-PAC-HYPO trial is a single-arm multi-center phase 2 study that evaluates the impact of a reminder app on the number of radiotherapy sessions with a bladder volume of < 200 mL during moderately hypo-fractionated radiotherapy for prostate cancer when compared to a historical control group also treated with moderately hypo-fractionated radiotherapy but not supported by an app. This control group is regarded as appropriate, since all patients were irradiated recently (2022–2024) with volumetric-modulated arc therapy (VMAT) at centers participating in the REFILL-PAC-HYPO trial. Moreover, the patients of the control group received Cone Beam Computed Tomography (CBCT) at each session. The time for the recruitment of all 27 patients will be 20 months. As the end of the 4-week course of radiotherapy represents the end of the study, the time for the REFILL-PAC-HYPO trial will be 21 months in total.

### 2.3. Eligibility Criteria

The criteria for inclusion and exclusion are given in Table 1.

### 2.4. Sample Size Calculation

The primary objectives of the REFILL-PAC-HYPO trial are the assessment of the effect of an app reminding prostate cancer patients treated with moderately hypo-fractionated radiotherapy to drink water before each treatment session on the number of sessions with bladder volumes of <200 mL and to demonstrate that this number is lower when compared to a historical control group not supported by a reminder app. To allow for a skewed distribution of the primary endpoint, the Wilcoxon–Mann–Whitney test will be applied for confirmatory statistical analysis. The calculation of the required sample size is based on an article by G.E. Noether [25]. In the external historical control group consisting of 52 patients, the mean number of radiation fractions with bladder volumes of <200 mL was 16.0 (SD 5.5), and the median number was 19.0 fractions (IQR 14.5–20.0) [13]. A decrease in this mean value by roughly 30% (to 11.2 fractions) is considered clinically relevant, based on the findings from our previous study and our clinical experience [13]. For illustrative purposes, translating this decrease into a non-parametric effect size framework (assuming, for simplicity, a normal distribution) leads to a probability of roughly 0.27 that the number of fractions of <200 mL with the reminder app will be larger than without the app. Based on this effect size, a sample size of 24 patients in the prospective REFILL-PAC-HYPO trial is required for the comparison with the historical control group to ensure a 90% power to reach statistical significance with a two-sided Wilcoxon Mann–Whitney-U-test and a 5% significance level. Assuming that roughly 10% of the enrolled patients will not be eligible for the primary analysis, since they may receive less than 20 fractions, a total number of 27 patients should be enrolled in the REFILL-PAC-HYPO trial.

## 3. Detailed Procedure

### 3.1. Assessments

The following characteristics are assessed before the start of the radiotherapy course: medical history, medication, age, body mass index, Karnofsky performance score, bladder volume, prostate volume, tumor stage, histology, Gleason score, level of prostate-specific antigen (PSA), risk group of prostate cancer, dose fractionation parameters, treatment volume, technique of radiotherapy, and patient’s smart phone experience and need for support. The corresponding timeline is given in Table 2.

### 3.2. Treatment and Interventions

#### 3.2.1. Moderately Hypo-Fractionated Radiotherapy

In the REFILL-PAC-HYPO trial, radiotherapy will be administered using moderate hypo-fractionation with 20 × 3.0 Gy over 4 weeks. The preferred technique for the radiation treatment of the prostate plus/minus seminal vesicles is VMAT. Moderately hypo-fractionated radiotherapy for prostate cancer may lead to adverse effects, including cystitis, proctitis, and skin reactions. In the case of grade 3 toxicity according to the Common Terminology Criteria for Adverse Events (CTCAE) version 5.0, radiotherapy may be delayed for up to 7 days without consequences [26]. If the time of delay is longer than 7 days, participation in the trial must be stopped and the principal coordinating investigator must be informed.

#### 3.2.2. Assessment of the Bladder Volume

Bladder volume is assessed by experienced staff members before the radiotherapy course during CT simulation and during each fraction of moderately hypo-fractionated radiotherapy using a CBCT. The staff members contour the urinary bladder, and its volume is calculated by the planning system.

#### 3.2.3. Mobile Application (Reminder App)

In the REFILL-PAC-HYPO trial, the patients are supported by a reminder app, which is developed by Nextlabel OHG from Lübeck. The purpose of the app is to remind the patients to drink 300 mL of water 45 min before each radiotherapy session. The time of the reminder can be set by the patients for each session.

#### 3.2.4. Questionnaire Regarding the App and Its Impact on Using Health Technology

At the end of the course of moderately hypo-fractionated radiotherapy, the patients will complete a questionnaire (modified according to [27,28]) regarding their satisfaction with the reminder app. If more than 20% of the patients are not satisfied, the app needs to be improved and requires modifications. If the dissatisfaction rate is >40%, the app is considered not useful. These thresholds were selected based on the protocols of previous trials by our group developed after discussions with the Ethic Committee responsible concerning this matter [29,30]. In addition, this questionnaire addresses the impact of participation in the trial, including the use of the reminder app, on the patient’s general attitude towards health technology.

### 3.3. Statistical Methods for Primary and Secondary Outcomes

The primary endpoint is defined as the number of radiotherapy sessions with bladder volumes of <200 mL at the end of the course of moderately hypo-fractionated radiotherapy (i.e., after 20 fractions). Descriptive measures of location and dispersion will be used to describe the results of the REFILL-PAC-HYPO trial. The impact of patient characteristics on the primary objective will be assessed using the Wilcoxon two-sample test. These characteristics include age (<75 vs. ≥75 years), Karnofsky performance score (70–80 vs. 90–100), body mass index (<30 vs. ≥30 kg/m^2^), prostate volume (<60 vs. ≥60 mL), pre-radiotherapy PSA level (<10 vs. ≥10 ng/mL), Gleason score (≤7 vs. 8–9), primary tumor stage (T1 vs. T2 or T3 [31]), risk group (low to intermediate vs. high), and antihormonal therapy before and/or during moderately hypo-fractionated radiotherapy (no vs. yes). The cohort of the REFILL-PAC-HYPO trial will be compared with the corresponding historical control group by a two-sided Wilcoxon–Mann–Whitney two sample test (significance level = 5%). A high degree of comparability between the prospective and retrospective patient data sets is expected. The potential heterogeneity of study populations is identified by comparing the above-mentioned characteristics using Wilcoxon–Mann–Whitney tests. Homogeneity is assumed if *p*-values are >0.20. Any factor indicating a tendency towards heterogeneity (*p* < 0.20) will be included in a multivariate count data Poisson regression model, including the number of radiotherapy sessions with bladder volumes of <200 mL as dependent variable. Corresponding characteristics and the binary factor (prospective cohort vs historical control group) are considered as independent variables. In the case of overdispersion, the Poisson model is replaced by a negative binomial model. In addition, a propensity score matching approach considering the above-mentioned patient characteristics will be applied to reduce the risk of selection bias.

Since no comparison with the historical control group is possible regarding the secondary objectives, these analyses focus only on descriptive analyses. The patient satisfaction results will be used to decide whether the reminder app needs modification. If the dissatisfaction rate is >20%, the app needs modifications. If the dissatisfaction rate is >40%, the app is considered not useful for improving the bladder filling status of patients treated with moderately hypo-fractionated radiotherapy for localized prostate cancer.

### 3.4. Data Protection

Data will be collected in accordance with the regulations set out in the Data Protection Act. All findings from the clinical trial will be stored on electronic data storage devices and treated with the utmost confidentiality. Organization measures will be taken in order to prevent the data from being communicated to unauthorized persons. Patients will only be identified via their individual patient numbers throughout the entire documentation and evaluation phase, and names or any information which would make the patient identifiable will not be used. For personal activation of the app for each study participant, Nextlabel OHG will receive the participant’s e-mail address. To ensure the protection of the participant’s e-mail address, a contract was concluded between the Sponsor (University Medical Center Schleswig-Holstein, UKSH) and Nextlabel OHG. This contract includes an approved data protection concept. Nextlabel will not have access to patient data that are not pseudonymized.

### 3.5. Data Management

Patient-related data are recorded pseudonymously, which means that a patient can be identified only by their unique number and their date of birth. A patient identification list is kept only in each center contributing patients to the REFILL-PAC-HYPO trial and will not be sent to the sponsor of the trial. For the collection and documentation of the data required for the REFILL-PAC-HYPO trial, specific forms are used.

Originals of the key documents of the REFILL-PAC-HYPO trial, including the documentation forms, will be kept by the sponsor of the trial for a period of at least 10 years after the completion of the final trial report. The principal investigator and the main investigator at each participating center, respectively, will keep administrative documents including correspondence with the responsible ethics committee and other authorities, patient identification lists, informed consent forms signed by the patients, copies of all documentation forms, and the general trial documents, including the study protocol, for at least 10 years after the completion of the final trial report. Original patient data need to be kept for the time period stipulated for the participating center, which is at least 10 years.

On-site monitoring is performed by the Centre for Clinical Trials, Lübeck, Germany, according to Good Clinical Practice and to the written standard operating procedures at the centers located in Germany. Visits include the initiation of the trial, visits during the period of the trial at intervals, which depend on the recruitment status and the quality of the data, and a close-out visit. Contributing centers outside Germany (e.g., in Denmark or Spain) are monitored in accordance with the national regulations in their own responsibility. Regular audits regarding the REFILL-PAC-HYPO trial are not planned. Since the trial does not meet the criteria for a study performed according to the German medicinal products act or medical device regulation, inspections by federal authorities are not scheduled.

The results of the REFILL-PAC-HYPO trial will be published in an international, peer-reviewed journal and are planned to be presented at scientific meetings or symposia. The corresponding reports and publications will be coordinated with the professional statistician involved. For these publications, the acronym REFILL-PAC-HYPO will be used.

## 4. Expected Results and Discussion

It is expected that the support provided by the reminder app will lead to a decrease in the mean value of radiation sessions with a bladder volume of <200 mL by 30% (to 11.2 sessions) when compared to the historical control group. Moreover, it is expected that the patients will be mostly satisfied with the reminder app, i.e., that the dissatisfaction rate will be below 20%. If this study shows that the reminder app helps to reduce the number of fractions with a bladder volume of <200 mL during moderately hypo-fractionated radiotherapy, a considerable number of patients with prostate cancer receiving this type of radiotherapy can benefit from such an app in the future.

Moderately hypo-fractionated radiotherapy lasting less than six weeks is becoming increasingly used for localized prostate cancer, since randomized trials have shown that this type of dose fractionation is not inferior to normo-fractionated radiotherapy (overall treatment time from 7.5 to 8 weeks) [4,5,6]. Only the CHHiP trial included patients with prostate cancers from all three risk groups, including high-risk tumors, which represented 12% of the trial cohort [4]. The other two trials were limited to intermediate-risk and low-risk tumors, respectively [5,6]. Thus, moderately hypo-fractionated radiotherapy is mainly used for patients with intermediate-risk and low-risk tumors. In two of the randomized trials, a dose fractionation regimen of 20 × 3.0 Gy over four weeks was used [4,5]. Like other fractionation regimens for prostate cancer, moderately hypo-fractionated radiotherapy can lead to radiation cystitis. Since the risk of grade ≥ 2 acute urinary toxicity, including radiation cystitis, is markedly increased if the bladder volume is <200 mL, it is very important to keep the bladder volume ≥ 200 mL during as many radiotherapy sessions as possible [8,10]. To improve bladder filling status during EBRT for prostate cancer, several studies have investigated the potential benefit of so-called drinking protocols requesting patients to drink a certain amount of water at a specific time before each radiotherapy session and before CT simulation [10,11,12,15,16,17,18,19,20,21,22,23,24,32]. However, following these protocols can be challenging for mostly elderly or even very elderly patients. The question of whether these patients may benefit from support by a reminder app has been raised. Such an app would remind patients to drink water at a pre-selected point in time. The REFILL-PAC-HYPO trial investigates an app reminding patients who are receiving moderately hypo-fractionated radiotherapy for localized prostate cancer to drink 300 mL of water 45 min before each treatment session. The trial evaluates whether this app reduces the number of fractions with a bladder volume of <200 mL during the radiotherapy course when compared to a historical control group of patients treated with moderately hypo-fractionated radiotherapy who were not supported by a reminder app.

Our previous study identified significant differences between patients with a pre-radiotherapy bladder volume of <200 mL and those with a volume of ≥200 mL regarding the number of fractions with a volume of <200 mL during a radiotherapy course of 20 fractions [13]. The mean and median numbers of fractions with a volume of <200 mL were 16.0 vs. 7.9 and 19.0 vs. 8.0, respectively. As a consequence, the REFILL-PAC-HYPO trial is limited to patients with a pre-radiotherapy bladder volume of <200 mL, since these patients are much more likely to benefit from the reminder app. For the comparison of the REFILL-PAC-HYPO cohort to the historical control group, a matching procedure is performed that considers nine patient- and tumor-related factors.

However, this study has several limitations that need to be considered when interpreting its results when they become available. Its single-arm design cannot exclude the Hawthorne effect, the small sample size may result in an insufficient statistical validity, and the historical control group may have unmeasured confounders due to retrospective data. The fact that patients in the REFILL-PAC-HYPO cohort must own a smartphone and be capable of using the app bears the risk of a selection bias. The urinary bladder is contoured by experienced staff members, and the bladder volume is automatically calculated by the planning system. However, the risk of an observer bias remains, since investigators know which patients use the reminder app. Blinding is not possible, since all patients participating in the prospective trial are supported by the app. In general, this lack of randomization could impact the trial’s internal validity.

## Figures and Tables

**Table 1:** Inclusion and exclusion criteria for the REFILL-PAC-HYPO trial.

Inclusion criteria	Histologically proven prostate cancer Indication for hypo-fractionated radiotherapy Possession of and ability to use a smart phone Bladder volume at CT simulation <200 mL Age ≥ 18 years Written informed consent Capacity of the patient to contract
Exclusion criteria	Radiotherapy of pelvic lymph nodes Expected non-compliance

**Table 2:** Timeline of enrolment, interventions, and assessments. ^#^ during each radiotherapy session.

	**Prior to** **radiotherapy**	**Weekly during the** **course of radiotherapy**	**At the end of radiotherapy**
Medical history and concomitant diseases	X		
Demographic data	X		
Concomitant medication	X		
Systemic anticancer treatment	X	X	X
Patient characteristics	X		
Performance status	X		X
Radiotherapy (given as planned ?)		X	X
Informed consent	X		
Assessment of adverse events and serious adverse events		X	X
**Bladder volume according to Cone-Beam Computed Tomography**	**X**	**X ^#^**	**X**
Experience with smartphones	X		
**Satisfaction with the reminder app**			**X**
**Impact of the reminder app on the use of health technology**			**X**

## Data Availability

Further information regarding this trial is available at clinicaltrials.gov (identifier: NCT06784115).

## Abbreviations

The following abbreviations are used in this manuscript:

BMI	Body Mass Index
CBCT	Cone Beam Computed Tomography
CT	Computed Tomography
CTCAE	Common Terminology Criteria for Adverse Events
EBRT	External Beam Radiotherapy
IQR	Interquartile Range
SD	Standard Deviation
VMAT	Volumetric-Modulated Arc Therapy
